# Accuracy of an Artificial Intelligence–Based Model for Estimating Leftover Liquid Food in Hospitals: Validation Study

**DOI:** 10.2196/35991

**Published:** 2022-05-10

**Authors:** Masato Tagi, Mari Tajiri, Yasuhiro Hamada, Yoshifumi Wakata, Xiao Shan, Kazumi Ozaki, Masanori Kubota, Sosuke Amano, Hiroshi Sakaue, Yoshiko Suzuki, Jun Hirose

**Affiliations:** 1 Department of Medical Informatics Institute of Biomedical Sciences Tokushima University Graduate School Tokushima Japan; 2 Division of Nutrition Tokushima University Hospital Tokushima Japan; 3 Department of Therapeutic Nutrition Institute of Biomedical Sciences Tokushima University Graduate School Tokushima Japan; 4 Health Information Management Center National Hospital Organization Kyushu Medical Center Fukuoka Japan; 5 Medical Information Technology Center Tokushima University Hospital Tokushima Japan; 6 Department of Oral Health Care Promotion Institute of Biomedical Sciences Tokushima University Graduate School Tokushima Japan; 7 foo.log Inc Tokyo Japan; 8 Department of Nutrition and Metabolism Institute of Biomedical Sciences Tokushima University Graduate School Tokushima Japan

**Keywords:** artificial intelligence, convolutional neural network, neural network, machine learning, malnourished, malnourishment, model, hospital, patient, nutrition, food consumption, dietary intake, diet, food intake, liquid food, nutrition management

## Abstract

**Background:**

An accurate evaluation of the nutritional status of malnourished hospitalized patients at a higher risk of complications, such as frailty or disability, is crucial. Visual methods of estimating food intake are popular for evaluating the nutritional status in clinical environments. However, from the perspective of accurate measurement, such methods are unreliable.

**Objective:**

The accuracy of estimating leftover liquid food in hospitals using an artificial intelligence (AI)–based model was compared to that of visual estimation.

**Methods:**

The accuracy of the AI-based model (AI estimation) was compared to that of the visual estimation method for thin rice gruel as staple food and fermented milk and peach juice as side dishes. A total of 576 images of liquid food (432 images of thin rice gruel, 72 of fermented milk, and 72 of peach juice) were used. The mean absolute error, root mean squared error, and coefficient of determination (*R^2^*) were used as metrics for determining the accuracy of the evaluation process. Welch *t* test and the confusion matrix were used to examine the difference of mean absolute error between AI and visual estimation.

**Results:**

The mean absolute errors obtained through the AI estimation approach were 0.63 for fermented milk, 0.25 for peach juice, and 0.85 for the total. These were significantly smaller than those obtained using the visual estimation approach, which were 1.40 (*P*<.001) for fermented milk, 0.90 (*P*<.001) for peach juice, and 1.03 (*P*=.009) for the total. By contrast, the mean absolute error for thin rice gruel obtained using the AI estimation method (0.99) did not differ significantly from that obtained using visual estimation (0.99). The confusion matrix for thin rice gruel showed variation in the distribution of errors, indicating that the errors in the AI estimation were biased toward the case of many leftovers. The mean squared error for all liquid foods tended to be smaller for the AI estimation than for the visual estimation. Additionally, the coefficient of determination (*R^2^*) for fermented milk and peach juice tended to be larger for the AI estimation than for the visual estimation, and the *R^2^* value for the total was equal in terms of accuracy between the AI and visual estimations.

**Conclusions:**

The AI estimation approach achieved a smaller mean absolute error and root mean squared error and a larger coefficient of determination (*R^2^*) than the visual estimation approach for the side dishes. Additionally, the AI estimation approach achieved a smaller mean absolute error and root mean squared error compared to the visual estimation method, and the coefficient of determination (*R^2^*) was similar to that of the visual estimation method for the total. AI estimation measures liquid food intake in hospitals more precisely than visual estimation, but its accuracy in estimating staple food leftovers requires improvement.

## Introduction

### Background

The prevalence of malnutrition among hospitalized patients is reportedly between 20% and 50% [1], and this rate is significantly high among patients who are older or who have cancer [2]. Malnourished hospitalized patients are at a higher risk of complications, such as pressure ulcers, infections [3], and frailty [4]. These are the risk factors of disability associated with daily living activities, and they can result in death [5,6]. In current superaged societies, malnutrition poses an increased risk. Therefore, an accurate evaluation of the nutritional status of hospitalized patients is crucial for the prevention of malnutrition among such patients [7].

Nutritional status is determined by anthropometric parameters (eg, body mass index) and laboratory parameters (eg, ion or protein concentration). Patients' food intake can also be used as an assessment metric because it affects their nutritional status [8]. Based on weight, the median plate waste in hospitals is 30% higher than that in other food service sectors [9]. Therefore, measurement and assessment of the actual amount of food consumed by patients are necessary.

The most accurate method for measuring food intake among hospitalized patients involves weighing foods before and after consumption [10]. Although this method optimizes accuracy, it is labor-intensive and requires space for holding soiled trays to measure waste [11]. In clinical environments, a popular method for evaluating food intake involves direct observation by medical staff. This approach is commonly referred to as the visual estimation method. However, it has been reported that the accuracy of the visual estimation method is lower than that of the weighing method [12,13], and the results obtained through these methods tend to vary depending on the training of the medical professionals and their job categories [14,15]. Additionally, although the measurement approach is simple, various problems exist, such as the fact that patients are often asked to measure their own food intake. This request is made because it is difficult for medical professionals to check all the food.

Recently, there have been significant advancements in the field of artificial intelligence (AI), and technological approaches for image analysis—such as organ segmentation [16] and lesion detection support [17]—have been utilized in various medical fields. Therefore, AI-based technological approaches can be applied to ensure improved accuracy in the measurement and evaluation of food intake among hospitalized patients. Additionally, such methods are more convenient than visual estimation methods because they estimate the remaining amount of food using digital images of food obtained through photography.

Currently, there exists an AI-based system that can estimate the classifications and names of foods through photographic images [18,19]. Additionally, Ege et al [20] proposed an AI-based system for estimating calories through the selection of recipes that match each food detected from photographic images. However, their proposed AI-based system estimates the caloric intake by identifying the predetermined menu based on photographic images of the meal before consumption. Therefore, there is no system that can be used to accurately measure and evaluate the actual amount of food consumed by considering the leftover amount.

### Objective

In this study, an AI-based model was developed that can be used to estimate the amount of leftover liquid food by learning the pattern of leftover liquid food obtained from images of liquid food in hospitals. There were three tasks associated with the estimation of leftovers from images of different foods. An object-detection approach was developed in this study for detecting multiple types of food on a tray and a classifier for determining the names of foods matching those in the detected object. Furthermore, the accuracy of the remaining task was evaluated because it pertains to the measurement and estimation of leftover liquid food.

## Methods

### Measurement of Leftover Liquid Food in Hospitals

**Figure 1:**
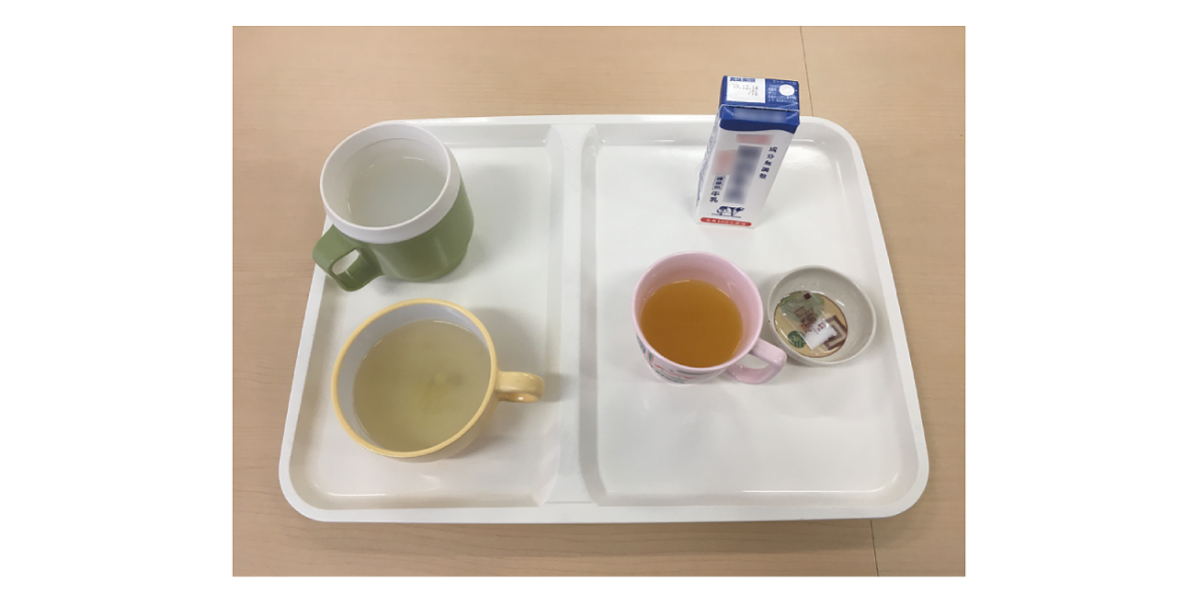
Example of liquid food served on a tray in hospitals.

Liquid foods were photographed to evaluate their leftovers (Figure 1). The liquid foods used in this study were similar to those provided to hospitalized patients, with multiple food items served on a tray.

The menu comprised a combination of staple food, side dishes, packaged beverages, and seasonings. The types of liquid foods are listed in Table 1. The leftover plates were evaluated through a measurement of the actual amount of each liquid food item on a digital scale, so that the leftovers of each liquid food item were on an 11-point scale ranging from 0 to 10 (Table 2).

**Table 1:** Types of dishes and number of images used for artificial intelligence (AI) training and evaluation.

Type of food and liquid food name		Training images, n	Evaluation images, n	Accuracy evaluation	
**Staple food**					
	Thin rice gruel	504	432	✓^a^	
**Side dishes 1**					
	Japanese clear soup	144	72		
	Vegetable soup	360	72		
	Miso soup	144	72		
	Red miso soup	66	6		
**Side dishes 2**					
	Fermented milk	72	72	✓	
	Peach juice	72	72	✓	
	Grape juice	72	72		
	Orange juice	72	72		
	Mixed juice	66	6		
	Fruit mix	66	6		
**Packaged beverage**					
	Milk	504	360		
	Milk for toddlers	66	6		
	Apple juice for toddlers	66	6		
	Orange juice for toddlers	66	6		
	Additive-free vegetable juice	66	6		
**Seasoning**					
	Salt	504	432		

^a^The checkmark indicates the liquid foods used for accuracy evaluation.

**Table 2:** Actual measurement of the converted values of the leftover liquid food.

Converted value	Leftover liquid food
0	Ingesting 5% or less of the entire amount.
1	Ingesting between 5% and 15% of the entire amount.
2	Ingesting between 15% and 25% of the entire amount.
3	Ingesting between 25% and 35% of the entire amount.
4	Ingesting between 35% and 45% of the entire amount.
5	Ingesting between 45% and 55% of the entire amount.
6	Ingesting between 55% and 65% of the entire amount.
7	Ingesting between 65% and 75% of the entire amount.
8	Ingesting between 75% and 85% of the entire amount.
9	Ingesting between 85% and 95% of the entire amount.
10	Ingesting 95% or more of the entire amount.

AI estimation was conducted by analyzing the liquid food images using an AI-based model for estimating leftover liquid food. All images of the lunch menu containing thin rice gruel, fermented milk, and peach juice were evaluated. Visual estimation was conducted by a person looking at similar liquid food images. Images were randomly selected from the images of the lunch menu containing rice gruel, fermented milk, and peach juice so that all the dishes with 0 to 10 leftovers of each dish were evaluated, and dietitians and students evaluated the same images. Each method used an 11-point scale to estimate the leftover liquid food. Visual estimation was performed by 10 dietitians from Tokushima University Hospital and 6 students from the Department of Medical Nutrition, Tokushima University. A total of 576 images of liquid food (432 images of thin rice gruel, 72 of fermented milk, and 72 of peach juice) were analyzed through AI estimation and visual estimation.

### Ethics Approval

This study was conducted as part of a study approved by the clinical research ethics committee at Tokushima University Hospital (#3758).

### Data Set

For a single menu, 12 types of liquid food images were created, each comprising the following portions: the state before eating (no. 1 in Table 3), in which the amount of leftover liquid food was 100%; 10 combinations of the states with some leftovers (nos. 2-11 in Table 3), in which the amounts of leftovers for each liquid food were at 0%, 10%, 20%, 30%, 40%, 50%, 60%, 70%, 80%, and 90%; and the state with no leftovers (no. 12 in Table 3), in which the amount of leftover liquid food was 0%.

**Table 3:** List of leftover liquid food combinations prepared for each grouping of dishes.

Number	Category	Staple food^a^	Side dishes 1^a^	Side dishes 2^a^
1	Before eating	10	10	10
2	Some leftovers	1	9	8
3	Some leftovers	3	8	6
4	Some leftovers	5	7	3
5	Some leftovers	7	6	1
6	Some leftovers	9	5	5
7	Some leftovers	0	4	2
8	Some leftovers	8	3	0
9	Some leftovers	6	2	7
10	Some leftovers	4	1	4
11	Some leftovers	2	0	9
12	No leftovers	0	0	0

^a^Converted values of the leftover liquid food.

**Figure 2:**
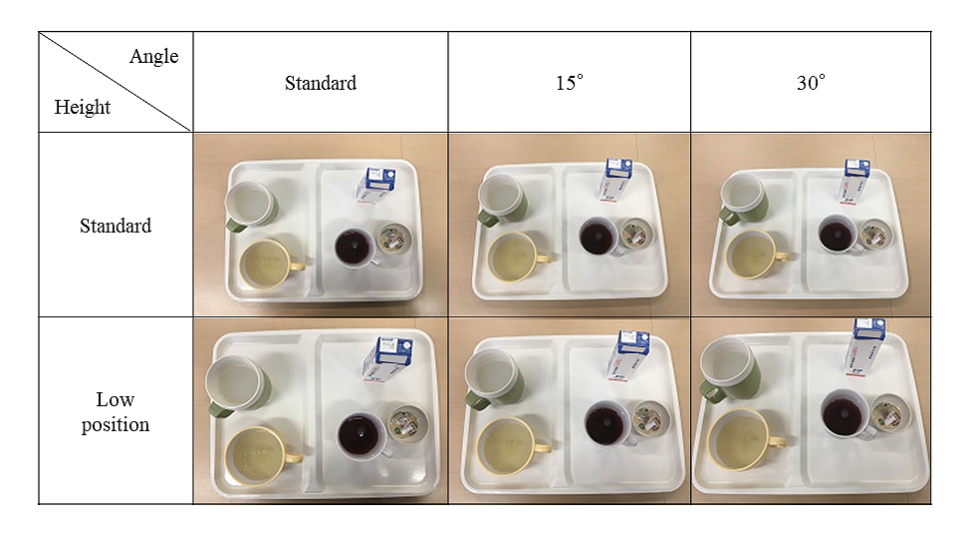
Photographs of a single portion of a single menu taken from six different camera positions.

For the camera position, the standard angle was the angle taken from directly above the liquid food tray at the height where the entire tray was contained, and the margin maintained (Figure 2). Angles of 15° and 30° were added to the standard angle. Additionally, the camera was repositioned to a lower position that included the entire tray and eliminated any blank space. Similarly, angles of 15° and 30° were added to the standard angles. A total of 6 different liquid food images were created for a single portion of a single menu.

In this study, liquid food images were taken separately for breakfast, lunch, and dinner on multiple dates and times, each under different conditions, such as light coming in from outdoors, for application in clinical environments. Images of the breakfast and dinner foods were used as the training images, and images of the lunch foods were used as the evaluation images. Therefore, the photographic environments for the training and evaluation images differed. The liquid foods used for accuracy evaluation are listed in Table 1.

### AI-Based Model for Estimating Leftover Liquid Food

A convolutional neural network (CNN), which is commonly applied in AI-based image analysis approaches, was used to analyze the liquid food images employed in this study. The AI-based model comprises two parts: (1) an object-detection part that identifies the positions of multiple dishes on a tray and extracts their regions from a single liquid food image and (2) a leftover-estimation part that classifies the names of liquid foods associated with the detected objects and estimates the amount of leftover liquid food. YOLOv3 [21] was used for object detection, following training using the FoodLog data set [22]. This is a one-class detection model with the liquid food region as the foreground and the others as the background. A multitask CNN was used to classify the names of liquid foods and estimate the leftover liquid food. Liquid food name classification is a task that consists of classifying 17 different liquid food names, and leftover estimation is a task that consists of classifying leftover liquid food on an 11-point scale. The architecture of the multitask CNN involved a calorie-volume estimation model based on the method proposed by Ege et al [23]. Both tasks were shared up to the last fully connected layer of ResNet50v2 [24], thereby resulting in 512-dimensional fully connected output layers for each task. The training process was fine-tuned using data prepared for this study through the ImageNet training model published by GluonCV [25] as the initial parameter. The loss function L for training was calculated as follows:



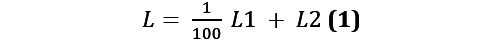



where L1 represents the cross-entropy loss for liquid food name classification, and L2 represents the cross-entropy loss for estimating the amount of leftover liquid food. The AI-based model development was performed using Python (version 3.6.5) as the programming language and PhpStorm and PyCharm as the integrated development environment.

### Accuracy Evaluation

The accuracies of the AI estimation and visual estimation methods were compared using actual measurements obtained through the weighing method employed for each staple food (thin rice gruel) and the side dishes (fermented milk and peach juice) as well as the total of these three liquid foods combined. The images of the side dishes created in different conditions for the training and evaluation processes were those of fermented milk, peach juice, grape juice, and orange juice. Fermented milk images, which had the lowest AI estimation accuracy, and peach juice images, which had the highest accuracy, were selected. Then, visual estimation was used to evaluate these images and those of the staple food (thin rice gruel).

In the hospital setting, liquid foods primarily contain milk, milk-based products including oatmeal, and clear liquid food [26]. In this study, menus that corresponded to these categories were selected. Thin rice gruel was selected because rice is often used in place of oatmeal in Japanese hospitals. Packaged beverages, salt, and seasonings were excluded from this study because it is difficult to evaluate such leftover foods through visual estimation.

Bland-Altman plots were used to examine the differences between the estimated and measured values and the limits of agreement were calculated as the mean difference ± 1.96 SD. The mean values of the measurements were calculated, and a paired *t* test was used to examine the differences.

There are two types of AI models: classification models, which are used to classify the category to which the objective variable belongs, and regression models, which are used to calculate the estimated value of the actual measured value. In this study, the estimated value of the continuous scale was used to estimate the amount of leftover liquid food, which is the average of the classification results achieved through multiple classification models. Because the AI-based model for estimating leftover liquid food predicts the estimated value of the actual measured value, mean absolute error, root mean squared error, and coefficient of determination (*R^2^*) were used as metrics for determining the accuracy of the evaluation process. The mean absolute error was calculated as follows:



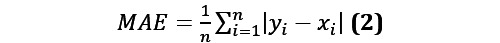



where x represents the estimated value, and y represents the measured value.

Welch *t* test was used to examine the differences between the AI estimation and visual estimation approaches in terms of the absolute error.

The root mean squared error squares the errors and then averages them, so that large errors are weighted more heavily. It is a useful metric when large errors are not particularly desirable. The root mean squared error was calculated as follows:



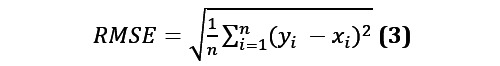



The coefficient of determination (*R^2^*) indicates the insignificance of the error compared to that of a model that always returns the average of the measured values. The closer the value is to 1, the higher its accuracy. It was used as a relative evaluation metric of which estimate was closer to the actual measurement—the AI estimation or the visual estimation. *R^2^* was calculated as follows:



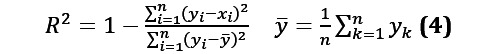



In addition, a confusion matrix of the estimated and measured values was created to evaluate the distribution of the absolute errors. The confusion matrix compares the measured values with the estimated values to evaluate which values have been incorrectly estimated. Statistical analyses were performed using SPSS Statistics version 24 (IBM Corp).

## Results

### Differences Between Estimated and Measured Values

**Figure 3:**
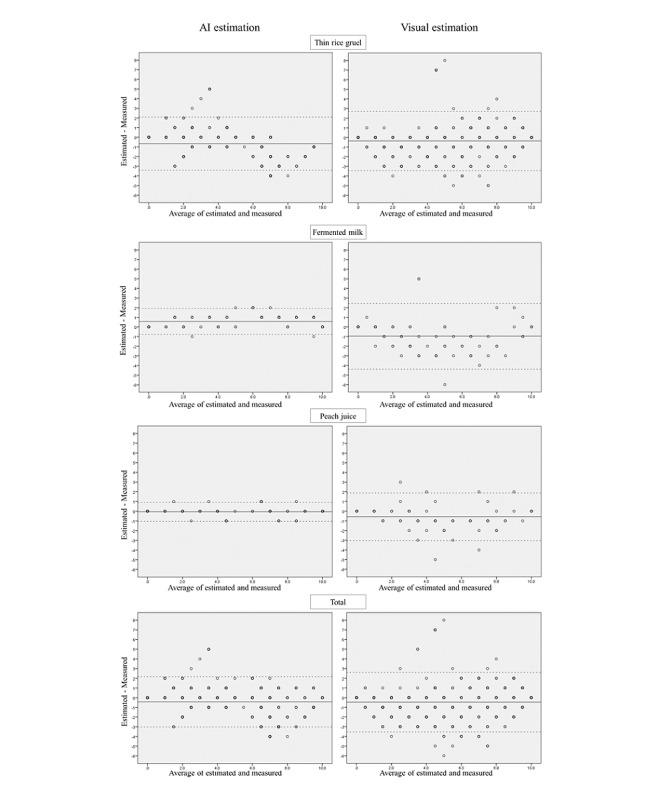
Bland-Altman analysis of the differences between estimated and measured values of leftover liquid food. AI: artificial intelligence.

The limits of agreement from the Bland-Altman plot estimated and measured values for AI estimation and visual estimation were −3.4 to 2.1 and −3.4 to 2.7 (thin rice gruel), −0.8 to 1.9 and −4.4 to 2.5 (fermented milk), −1.0 to 0.9 and −3.0 to 1.9 (peach juice), −3.0 to 2.2 and −3.5 to 2.6 (total) (Figure 3). The differences between the estimated and measured values by AI estimation for fermented milk and peach juice were particularly small. The value of the measurements by AI estimation for peach juice was not significantly different from the estimated value (4.53) and the measured value (4.58) (Table 4). The estimated value by AI for fermented milk (5.15) was significantly larger than the measured value (4.58). For the rest, the estimated value was significantly smaller than the measured value.

**Table 4:** Comparison of estimated and measured values of leftover liquid food.

	Leftover food, n	Measured value	AI^a^ estimation		Visual estimation	
			Estimated value	*P* value	Estimated value	*P* value
Thin rice gruel	432	4.58	3.93	<.001	4.21	<.001
Fermented milk	72	4.58	5.15	<.001	3.62	<.001
Peach juice	72	4.58	4.53	.35	4.01	<.001
Total	576	4.58	4.15	<.001	4.11	<.001

^a^AI: artificial intelligence.

### Mean Absolute Error

The mean absolute error of staple food leftovers obtained using the AI estimation approach (0.99) was not significantly different from that obtained via visual estimation (0.99) (Table 5). Moreover, the mean absolute errors obtained through the AI estimation approach for side dishes were 0.63 for fermented milk and 0.25 for peach juice. These were significantly smaller than those obtained using the visual estimation approach for fermented milk (1.40) and peach juice (0.90). The total mean absolute error obtained through AI estimation (0.85) was also significantly smaller than that obtained through visual estimation (1.03).

**Table 5:** Mean absolute errors obtained using the AI^a^ estimation and visual estimation methods.

	Images, n	AI estimation	Visual estimation	*P* value
Thin rice gruel	432	0.99	0.99	.96
Fermented milk	72	0.63	1.40	<.001
Peach juice	72	0.25	0.90	<.001
Total	576	0.85	1.03	.009

^a^AI: artificial intelligence.

### Root Mean Squared Error

The root mean squared error tended to be smaller for the AI estimation of thin rice gruel (1.55), fermented milk (0.89), peach juice (0.50), and total (1.39) than that for the visual estimation of thin rice gruel (1.61), fermented milk (1.98), peach juice (1.37), and total (1.64) (Table 6).

**Table 6:** Root mean squared error obtained using the AI^a^ estimation and visual estimation methods.

	Images, n	AI estimation	Visual estimation
Thin rice gruel	432	1.55	1.61
Fermented milk	72	0.89	1.98
Peach juice	72	0.50	1.37
Total	576	1.39	1.64

^a^AI: artificial intelligence.

### Coefficient of Determination

The coefficient of determination (*R^2^*) for staple foods tended to be smaller for the AI estimation method (0.69) than for the visual estimation (0.78) method. However, the coefficient of determination (*R^2^*) for side dishes tended to be larger for the AI estimation of fermented milk (0.94) and peach juice (0.98) than that for the visual estimation of fermented milk (0.62) and peach juice (0.82) (Table 7). The *R^2^* value for the total was equal in terms of accuracy between the AI estimation (0.78) and visual estimation (0.77) methods.

**Table 7:** Coefficient of determination (*R^2^*) for the AI^a^ estimation and visual estimation methods.

	Images, n	AI estimation	Visual estimation
Thin rice gruel	432	0.69	0.78
Fermented milk	72	0.94	0.62
Peach juice	72	0.98	0.82
Total	576	0.78	0.77

^a^AI: artificial intelligence.

### Distribution of Errors

**Figure 4:**
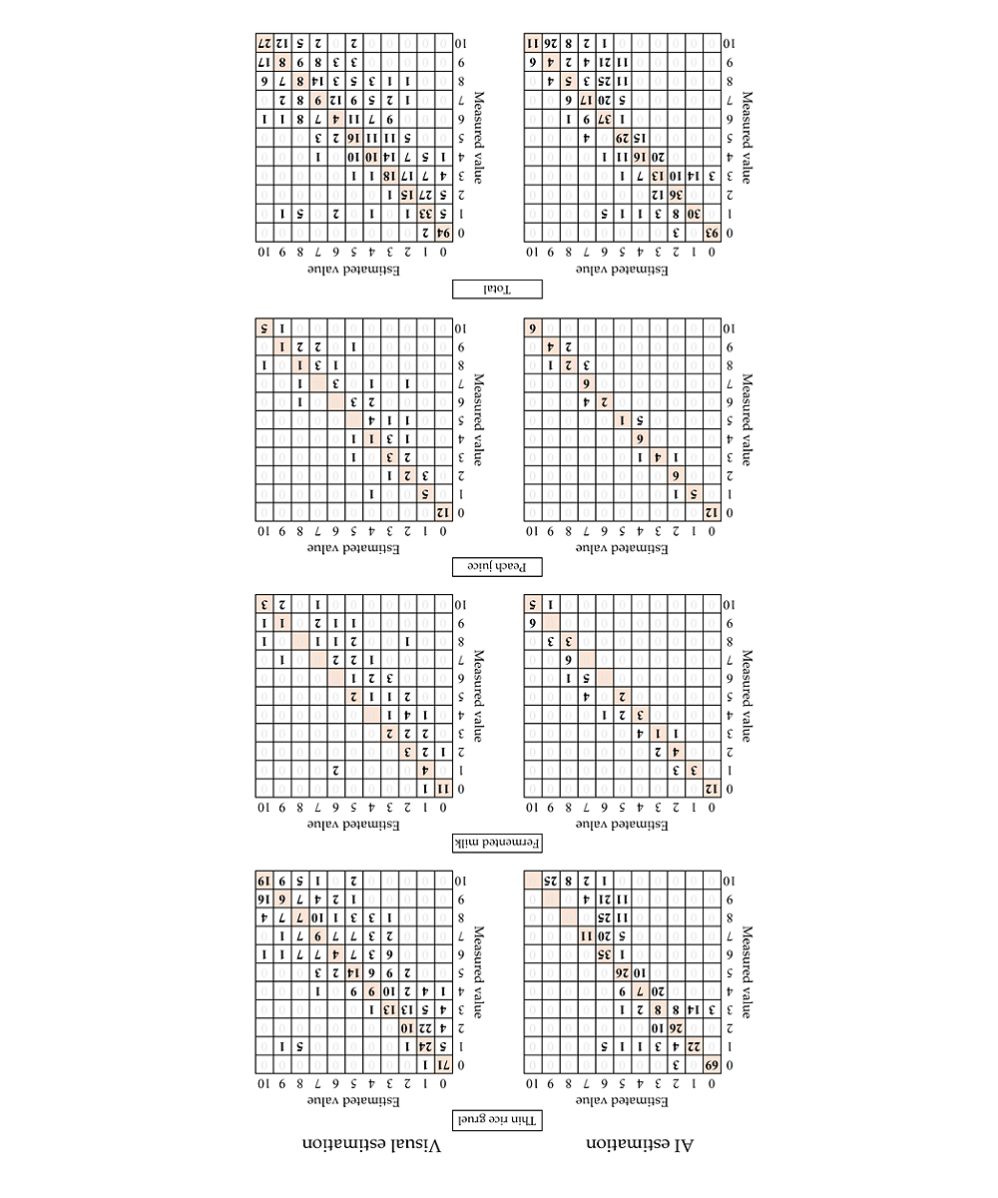
Confusion matrices of the estimated and measured values. AI: artificial intelligence.

The confusion matrix for staple foods (Figure 4) shows variation in the distribution of errors, indicating that the errors in the AI estimation were biased toward the case of many leftovers. The values converged to a specific estimated value, as the estimated values were biased toward 6 for images with measured values of 6 to 9. In addition, many evaluations estimated that the estimated value was less than the measured value for both the AI estimation and visual estimation methods. However, for the confusion matrix of side dishes, the AI estimation had a small error, and the estimated and measured values were in close agreement, whereas the visual estimation demonstrated a large variability. The confusion matrix for the total also showed the same trend as for the staple food, with more evaluations estimating that the leftover was less than the measured value.

## Discussion

### Principal Findings

The AI estimation approach achieved a smaller mean absolute error and root mean squared error and a larger coefficient of determination (*R^2^*) than the visual estimation approach for the side dishes. Additionally, the AI estimation approach achieved a smaller mean absolute error and root mean squared error compared to the visual estimation method, while the coefficient of determination (*R^2^*) was similar to that of the visual estimation method for the total. These results indicate that the accuracy of the AI estimation method was high, except for staple foods. In particular, peach juice was highly reliable because there was no difference between the AI estimation and the weighing method. Underestimating liquid food consumption can lead to incorrect nutritional guidance, whereas a correct assessment of food intake can lead to improvement through nutritional intervention.

The accuracy of estimation through the AI-based model was evaluated by comparing the estimated value to that of the actual measured value using the weighing method. For the accuracy indicator of the continuous scale, it is recommended to use the mean absolute error and the root mean squared error when evaluating the prediction performance of the same scale and applying measures, such as the coefficient of determination (*R^2^*), when outliers are included [27]. Therefore, three indicators—the mean absolute error, the root mean squared error, and the coefficient of determination (*R^2^*)—were used in this study. On the other hand, previous studies of human visual estimation of photographed food images have used mean differences as the accuracy indicator [28]. The analysis has been reported to be highly reliable for visual estimation using food images because it is highly correlated with the actual value obtained via the weighing method.

The visual estimation approach used in this study was as accurate as the visual estimation method used in previous studies. The AI estimation approach achieved higher accuracy than the visual estimation approach, suggesting that the AI estimation approach is more reliable for the precise measurement of liquid food intake. Moreover, the mean absolute error achieved through the AI estimation method was 8.5% in this study, indicating that the goal of this measurement method in clinical contexts was also achieved because the measurement method used in clinical contexts should have an error of less than 10% using the weighing method [29].

Regarding the side dishes, the AI estimation approach had a small error and was in close agreement with the measured values (Figure 4). The value of the coefficient of determination (*R^2^*) was also large, but it was smaller for staple foods. However, there was no difference in the mean absolute error. These results suggest that a large percentage of AI estimators made evaluations that had large errors. The confusion matrix shows that estimates for images with actual values ranging between 6 and 9 were biased toward 6, and the image features for distinguishing between 6 and 9 were not well discovered during the training process. For staple foods, the fact that the error grew larger when there was a large amount of leftover liquid foods remains an issue. In this study, liquid foods were prepared such that the number of cases per leftover would be equal, to make it easier to discern the accuracy of each leftover. However, in a previous study conducted in a clinical environment, the mean value of food intake was 82.5% [15]. Therefore, it is conceivable that the accuracy of the AI estimation could be even higher in actual clinical environments because there is less leftover food.

Liquid foods are recognized via the information obtained from the image, such as its color, whether it is well-lit, and its density [19]. In this study, the color and density of the liquid food were ascertained from this information. The fact that the accuracy levels achieved through AI estimation varied significantly among different liquid food types suggests that the estimation was affected by differences in color between the liquid food and the dish and the density of the liquid food. In this study, dishes that were actually served to patients in hospital wards were used, assuming a demonstration in clinical contexts. The thin rice gruel was pale white, and the dishes were white, thus similar in color. Furthermore, it was difficult to distinguish the border between liquid food and dishes because thin rice gruel is translucent and thick. This attribute may be the reason for the lower accuracy obtained compared to that of fermented milk, which is similar in color. Therefore, the accuracy of AI estimation for thin rice gruel could be improved by changing the color of the dish to a non-white color.

### Limitations

There are four limitations of this study. First, images of hospital liquid food taken using a camera were used for the visual estimation process to compare it with the AI estimation process. In clinical contexts and environments, medical staff estimate and record dietary intake by looking at the actual food. Therefore, it is also necessary to compare the results of the visual estimation approach by ensuring that medical staff look at the actual foods provided to patients and compare the results with those achieved through the AI estimation of food images taken in wards. Second, packaged beverages were excluded from this study because it is difficult to evaluate leftover liquid foods through visual estimation. For such foods, it is necessary to consider methods such as measuring by transferring the leftover liquid food to another dish. Third, this study is limited to the evaluation of liquid food images in a single institution. Because the menus and plates of liquid foods served to patients vary from institution to institution, it is necessary to evaluate whether the training images used in this study can be used to estimate the amount of leftover liquid food in multiple institutions while determining the additional training images required for each. Finally, the usability of the proposed AI-based measurement method is unclear. In daily use, systems that use image analysis to support food recording have been evaluated for their usability [22]. In clinical contexts and environments, further research is required to evaluate whether the use of AI-based measurement methods can be easily executed by medical staff.

### Conclusions

The proposed AI-based model demonstrated improved accuracy in the measurement and evaluation of leftover side dishes and similar accuracy levels for the total leftovers compared to the visual estimation method for leftover liquid foods. Additionally, errors incurred in the AI estimation approach were within the acceptable range of the weighing method, thereby indicating that the proposed AI-based model for estimating the amount of leftover liquid food can be applied in clinical contexts and environments. However, further evaluations and improvements of the AI-based model presented in this study are necessary for the development of an AI estimation method that can be used to accurately measure the intake of liquid food in hospitals.

## Abbreviations

AI: artificial intelligence
CNN: convolutional neural network

## References

[1] Norman K, Pichard C, Lochs H, Pirlich M (2008). Prognostic impact of disease-related malnutrition. Clin Nutr.

[2] Pirlich M, Schütz T, Norman K, Gastell S, Lübke HJ, Bischoff SC, Bolder U, Frieling T, Güldenzoph H, Hahn K, Jauch K, Schindler K, Stein J, Volkert D, Weimann A, Werner H, Wolf C, Zürcher G, Bauer P, Lochs H (2006). The German hospital malnutrition study. Clin Nutr.

[3] Correia MITD, Hegazi RA, Higashiguchi T, Michel J, Reddy BR, Tappenden KA, Uyar M, Muscaritoli M (2014). Evidence-based recommendations for addressing malnutrition in health care: an updated strategy from the feedM.E. Global Study Group. J Am Med Dir Assoc.

[4] Boulos C, Salameh P, Barberger-Gateau P (2016). Malnutrition and frailty in community dwelling older adults living in a rural setting. Clin Nutr.

[5] Söderström L, Rosenblad A, Thors Adolfsson E, Bergkvist L (2017). Malnutrition is associated with increased mortality in older adults regardless of the cause of death. Br J Nutr.

[6] Hsu Y, Chou M, Chu C, Liao M, Wang Y, Lin Y, Chen L, Liang C (2019). Predictive effect of malnutrition on long-term clinical outcomes among older men: a prospectively observational cohort study. J Nutr Health Aging.

[7] Donini LM, Scardella P, Piombo L, Neri B, Asprino R, Proietti AR, Carcaterra S, Cava E, Cataldi S, Cucinotta D, Di Bella G, Barbagallo M, Morrone A (2013). Malnutrition in elderly: social and economic determinants. J Nutr Health Aging.

[8] Agarwal E, Ferguson M, Banks M, Bauer J, Capra S, Isenring E (2012). Nutritional status and dietary intake of acute care patients: results from the Nutrition Care Day Survey 2010. Clin Nutr.

[9] Williams P, Walton K (2011). Plate waste in hospitals and strategies for change. e-SPEN, the European e-Journal of Clinical Nutrition and Metabolism.

[10] Kirkpatrick SI, Subar AF, Douglass D, Zimmerman TP, Thompson FE, Kahle LL, George SM, Dodd KW, Potischman N (2014). Performance of the automated self-administered 24-hour recall relative to a measure of true intakes and to an interviewer-administered 24-h recall. Am J Clin Nutr.

[11] Connors PL, Rozell SB (2004). Using a visual plate waste study to monitor menu performance. J Am Diet Assoc.

[12] Husted MM, Fournaise A, Matzen L, Scheller RA (2017). How to measure energy and protein intake in a geriatric department - A comparison of three visual methods. Clin Nutr ESPEN.

[13] Amano N, Nakamura T (2018). Accuracy of the visual estimation method as a predictor of food intake in Alzheimer's patients provided with different types of food. Clin Nutr ESPEN.

[14] Palmer M, Miller K, Noble S (2015). The accuracy of food intake charts completed by nursing staff as part of usual care when no additional training in completing intake tools is provided. Clin Nutr.

[15] Kawasaki Y, Sakai M, Nishimura K, Fujiwara K, Fujisaki K, Shimpo M, Akamatsu R (2016). Criterion validity of the visual estimation method for determining patients' meal intake in a community hospital. Clin Nutr.

[16] Roth H (2015). Deep convolutional networks for pancreas segmentation in CT imaging. SPIE Medical Imaging.

[17] Setio AAA, Ciompi F, Litjens G, Gerke P, Jacobs C, van Riel SJ, Wille MMW, Naqibullah M, Sanchez CI, van Ginneken B (2016). Pulmonary nodule detection in CT images: false positive reduction using multi-view convolutional networks. IEEE Trans. Med. Imaging.

[18] Anthimopoulos MM, Gianola L, Scarnato L, Diem P, Mougiakakou SG (2014). A food recognition system for diabetic patients based on an optimized bag-of-features model. IEEE J Biomed Health Inform.

[19] Mezgec S, Koroušić Seljak Barbara (2017). NutriNet: a deep learning food and drink image recognition system for dietary assessment. Nutrients.

[20] Ege T, Yanai K (2017). Estimating food calories for multiple-dish food photos.

[21] Redmon J, Frhadi A (2018). YOLOv3: an incremental improvement. Computer Vision and Pattern Recognition.

[22] Aizawa K, Maeda K, Ogawa M, Sato Y, Kasamatsu M, Waki K, Takimoto H (2014). Comparative study of the routine daily usability of FoodLog: a smartphone-based food recording tool assisted by image retrieval. J Diabetes Sci Technol.

[23] Ege T, Yanai K (2017). Simultaneous estimation of food categories and calories with multi-task CNN.

[24] He K, Zhang X, Ren S, Sun J (2016). Deep Residual Learning for Image Recognition.

[25] Model zoo classification. GluonCV.

[26] DeWitt TM (2015). An exploratory study: clinical dietitians do not view the full liquid diet as best practice for the post-operative patient. J Nutr Food Sci.

[27] Shcherbakov MV, Brebels A, Shcherbakov NL, Tyukov AP (2013). A survey of forecast error measures. World Applied Sciences Journal (Information Technologies in Modern Industry, Education & Society).

[28] Williamson DA, Allen HR, Martin PD, Alfonso AJ, Gerald B, Hunt A (2003). Comparison of digital photography to weighed and visual estimation of portion sizes. J Am Diet Assoc.

[29] Sharma M, Rao M, Jacob S, Jacob CK (1998). Validation of 24-hour dietary recall: a study in hemodialysis patients. J Ren Nutr.

